# Synthesis and Characterization of Luminescent and Antibacterial Europium-Titanate nanotubes

**DOI:** 10.1021/acsomega.5c08930

**Published:** 2025-12-13

**Authors:** Enzo O. Borazo, Rodolpho A. N. Silva, Gabriel L. Colombo, Ana M. Pires, Emilson R. Viana, Gustavo H. Couto, Renata D. Adati, Cristiane Pilissão

**Affiliations:** † Department of Chemistry and Biology, Federal University of Technology Parana (UTFPR), Curitiba 81280-340, PR, Brazil; ‡ Department of Sustainable Development and Ecological Transition, 19050 University of Eastern Piedmont “A. Avogadro”, Vercelli 13100, Italy; § Department of Analytical, Physical-Chemistry and Inorganic Chemistry, Sao Paulo State University (UNESP), Institute of Chemistry, Araraquara 01049-010, SP, Brazil; ∥ Department of Chemistry and Biochemistry, Sao Paulo State University (UNESP), School of Science and Technology, Presidente Prudente 19060-900, SP, Brazil; ⊥ Department of Physics, Federal University of Technology Parana (UTFPR), Curitiba 80230-901, PR, Brazil

## Abstract

Antibacterial resistance poses a growing threat to public health by reducing the effectiveness of conventional antibiotics. Nanohybrid materials, such as titanate nanotubes (TiNts), represent a promising alternative owing to their low cost, chemical stability, and biocompatibility. In this study, we functionalized TiNts with europium ions (Eu^3+^) to enhance both optical properties and antibacterial activity. The incorporation of Eu^3+^ extended the material’s absorption from the ultraviolet into the visible region. Two luminescent hybrids were obtained: (i) TiNts/Eu, prepared by Eu^3+^ adsorption onto nanotubes, synthesized via an alkaline hydrothermal treatment, and (ii) TiNts­[Eu­(tta)_3_phen], produced through coordination of Eu^3+^ ions with the TiNts and the organic ligands thenoyltrifluoroacetone (tta) and 1,10-phenanthroline (phen). TiNts/Eu displayed the characteristic red emission of Eu^3+^, arising from the ^5^D_0_ → ^7^F_*J*_ (*J* = 0–4) transitions, confirming efficient energy transfer from both the inorganic host and organic ligands. The introduction of tta and phen further improved the material’s light absorption in the UV region (200–400 nm). Antibacterial assays revealed inhibition zones of 5 and 11 mm against *Staphylococcus aureus* and *Escherichia coli*, respectively, for TiNts/Eu. In contrast, the fully coordinated hybrid TiNts­[Eu­(tta)_3_phen] exhibited enhanced antibacterial activity, with inhibition zones of 14 mm. These findings highlight Eu^3+^ functionalized titanate nanohybrids as highly potent antimicrobial agents and versatile luminescent materials for biomedical applications.

## Graphical abstract

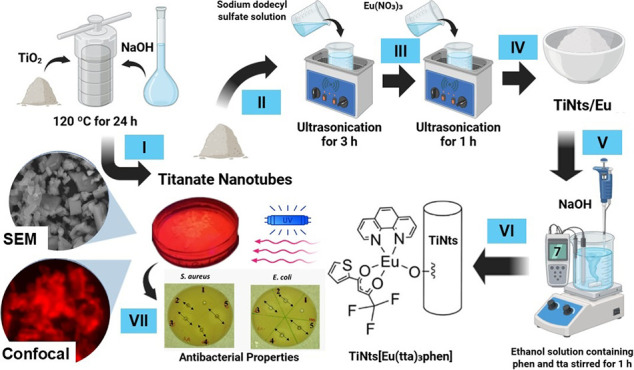

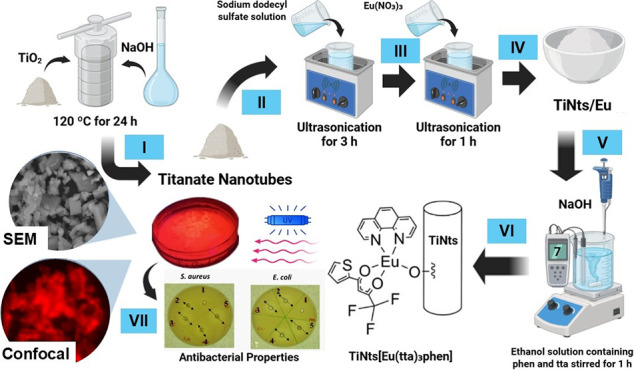

## 1 Introduction

Bacterial infections remain a significant global health concern. A key factor exacerbating this issue is the rapid rise of antimicrobial resistance (AMR), which compromises the effectiveness of conventional treatments. AMR contributes to increased healthcare costs and is estimated to cause approximately 700,000 deaths annually.^1,2^ In recognition of its severity, the World Health Organization has identified AMR as one of the most urgent public health threats worldwide.^3^ The emergence and spread of AMR are driven by multiple factors, including the inappropriate use of antimicrobial agents in human and veterinary medicine, inadequate infection control practices, poor sanitation, and unsafe food handling.^2,4–6^ As a result, several bacterial families have been classified as multidrug-resistant organisms posing significant challenges to healthcare systems. Notable examples include *Acinetobacter*, *Pseudomonas*, and members of the *Enterobacteriaceae* family, such as *Klebsiella pneumoniae*, *Escherichia coli*, and *Enterobacter* species.^7^ These pathogens are frequently resistant to multiple classes of antibiotics and are associated with severe, often life-threatening infections, including bloodstream infections and pneumonia.^8^

Despite significant progress in antimicrobial therapies, the rapid emergence of resistant bacterial strains highlights the urgent need for alternative antibacterial strategies and innovative antimicrobial materials.^9,10^ In this context, nanohybrid and nanoscale materials, particularly metal and metal oxides, have gained attention as promising candidates due to their multifunctional character and tunable physicochemical properties, which allow enhanced antibacterial performance beyond that of individual components.^3,11–13^ Among these, titanium dioxide-based nanomaterials stand out for their unique combination of biocompatibility and low cytotoxicity, and potent antimicrobial activity, positioning them strong candidates for next-generation antibacterial agents.^14–16^

Titanium dioxide (TiO_2_), especially in the form of titanate nanotubes (TiNts), have emerged as promising materials owing to low cost, environmental compatibility, chemical and mechanical stability, high surface area, and strong oxidative potential. As a wide-band gap semiconductor (3.0–3.4 eV), TiO_2_ is mainly active under near-ultraviolet (UV) light, limiting its visible-light efficiency. To address this, TiO_2_ is often doped with luminescent elements to enhance visible-light absorption and improve photocatalytic performance.^17,18^ Additionally, TiNts are particularly valuable because their high ion-exchange capacity allows for intercalation of various metal ions and surface modification through hydroxyl groups, enabling tunable optical and electronic properties without compromising the nanotubular structure.^19,20^ Lanthanide ions, typically trivalent, are especially effective dopants due to their unique electronic configurations, low toxicity, and antibacterial properties. Their strong coordination ability with inorganic and organic ligands facilitates the formation of diverse coordination compounds, broadening TiNts’s functional applications in areas such as photoluminescence and biomedicine.^21,22^

Europium (Eu^3+^) ions are recognized for their strong photoluminescent properties in the visible region, making them excellent candidates for extending the photoresponse of TiO_2_-based materials.^23,24^ Under UV excitation, Eu^3+^ ions emit characteristic red photoluminescence. However, their inherently low absorption cross-section often results in weak fluorescence unless they are coordinated with suitable host ligands.^25,26^ TiO_2_, in turn, provides a suitable host matrix for lanthanides incorporation, offering a robust platform for the development of luminescent hybrid materials.^27–29^ Zhao *et al*. reported that europium (Eu^3+^) doping in TiO_2_ introduces Impurity Energy Levels (IELs) located in the middle of the band gap. These IELs enable two-step electronic transitions from the valence band to the conduction band via the impurity states, enhancing visible light absorption above 390 nm. The octahedral crystal field allows intraband transitions within the Eu^3+^ *4f* states, further improving optical absorption.^30–34^

However, the incorporation of Eu^3+^ into TiNts modifies their electronic and optical properties while enhancing antimicrobial activity through increased reactive oxygen species (ROS) generation and improved charge separation.^30^ For example, H. Gujjaramma and coauthors^27^ developed Eu^3+^-doped inorganic nanostructures for photocatalytic and antibacterial studies. Tests were carried out against both Gram-positive and Gram-negative bacteria, and the results demonstrated the versatility of the material in inhibiting the growth of both cultures. The antimicrobial activity was explained in terms of the interaction between the material and the bacterial cell membrane, ultimately leading to cell death.^31^

In this work, two synthetic strategies were investigated to obtain Eu^3+^ based luminescent hybrids with titanate nanotubes. In the first approach, Eu^3+^ ions are adsorbed onto TiNts synthesized via an alkaline hydrothermal process, resulting in a luminescent hybrid TiNts/Eu. In the second, Eu^3+^ ions were coordinated to both titanate nanotubes and the organic ligands thenoyltrifluoroacetone (tta) and 1,10-phenanthroline (phen), producing the luminescent hybrid TiNts­[Eu­(tta)_3_phen]. The Eu^3+^ complex [Eu­(tta)_3_phen] is promising for both biomedical and industrial applications.^26^ In biomedicine, it enables sensitive time-gated imaging and targeted biosensing. Industrially, its stability and efficiency support its use in anticounterfeiting inks, LED phosphors, optical data storage, and UV dosimetry. To further enhance photophysical and antibacterial performance against *Staphylococcus aureus* and *Escherichia coli*, Eu^3+^ ions were incorporated into titanate nanotubes (TiNts), combining the matrix’s high surface area and ion-exchange capacity with luminescent and antibacterial activities. This synergy yields a multifunctional material for diverse applications.

## 2 Materials and Methods

### 2.1 Materials and Instrumentation

All reagents were of analytical grade and used without further purification. Europium nitrate solutions Eu­(NO_3_)_3_ were prepared by dissolving Eu_2_O_3_, 99.9%, Sigma-Aldrich, in concentrated nitric acid, followed by dilution with distilled water. The ligands 1,10-phenantroline (phen, 99.99%), and thenoyltrifluoroacetone (tta 99%), were purchased from Sigma-Aldrich, while Aeroxide TiO_2_ P25 powder was obtained from Evonik Industries AG (Brazil). *Escherichia coli* ATCC 25922 and *Staphylococcus aureus* ATCC 25923 were kindly provided by the Biomass and Bioenergy Research Laboratory (LAPREBB, PR, Brazil) through the Tropical Cultures Collection of the André Tosello Foundation (Campinas, Brazil). Other commercial reagents were of analytical grade, and used without prior purification.

Fourier transform infrared (FTIR) spectra were recorded using KBr pellets. Measurements were performed on a Bruker Alpha II FTIR spectrometer in transmission mode over the range of 4000–400 cm^–1^, with a resolution of 4 cm^–1^, using a Bomem spectrophotometer. The morphology of the samples was examined using a Carl Zeiss EVO MAIS scanning electron microscopy (SEM), equipped with energy-dispersive X-ray spectroscopy (EDS) for qualitative chemical analysis. EDS measurements were performed using an EDSX-Max 20 mm^2^ detector and a WDS Inca Wave 500 system to assess the elemental composition of the synthesized materials. Transmission electron microscopy (TEM) analyses were performed using a JEOL JEM-1200EX II instrument, which provides morphological characterization with a spatial resolution of approximately 0.5 nm. To prepare the samples, they were homogenized in ethanol or water in an ultrasound bath and dripped onto a 300-mesh copper screen with Formvar/carbon film. X-ray diffraction (XRD) patterns were recorded using a D2 PHASER diffractometer equipped with a Cu Kα radiation source (sealed tube), nickel filter, and LYNXEYE detector. Data were collected in Bragg–Brentano geometry over a 2θ range of 5–80°, with a step size of 0.02° and a counting time of 0.6 s per step.

Excitation and emission spectra, as well as luminescence decay curves of the europium complexes in powder form, were recorded at room temperature using a Horiba-Jobin Yvon Fluorolog-3 FL3–22 spectrofluorometer (IQ-UNESP) equipped with a Hamamatsu R928P photomultiplier. A 450 W continuous xenon short arc lamp (UXL-450S-O, USHIO INC.) was used for excitation and emission spectra measurements, while luminescence decay curves were obtained using a 0.15 J per flash high-stability short arc xenon flashlamp (FX-1102, Excelitas Technologies) with an initial delay of 0.05 ms. Experimental parameters were as follows: excitation wavelength 377.5 nm, excitation slit 1.0 nm, emission slit 1.0 nm, excitation grating 1200/330, emission grating 1200/500, and integration time 0.1 s. Luminescence lifetime measurements employed a flash duration of 41 ms over 50 flashes. The luminescence decay curves of the samples were analyzed using a biexponential fitting model, which provided the best agreement with the experimental data.

For systems exhibiting biexponential decay, the average lifetime (τ) was estimated using eq 1:^35^

1 $$\tau =\frac{A_{1}{\tau_{1}}^{2}+A_{2}{\tau_{2}}^{2}}{A_{1}\tau_{1}+A_{2}\tau_{2}}$$

where *A*_1_ and *A*_2_ are the amplitudes of the decay components, and τ_1_ and τ_2_ are the corresponding values for each element.

To evaluate the photostability of the synthesized systems, the emission intensity of the Eu^3+^ ^5^D_0_ → ^7^F_2_ transition (615 nm) was continuously monitored over a period of 1 h. Excitation was set at 393 nm (corresponding to the Eu^3+^ ^5^L_6_ ← ^7^F_0_ transition) for the TiNts/Eu system, and at 377 nm (ligand absorption band) for the [Eu­(tta)_3_(phen)] complex and the TiNts­[Eu­(tta)_3_phen] hybrid.

Confocal laser scanning microscopy (CLSM) was used to evaluate the fluorescence properties of europium (Eu^3+^) adsorbed onto titanate nanotubes (TiNts/Eu) and europium (Eu^3+^) coordinated with TiNts, tta, and phen-TiNts­[Eu­(tta)_3_phen]. Analyses were performed on an Olympus FV1200 microscope. Samples were excited using a 405 nm laser, and emission was detected using an SDM 490 dichroic mirror combined with a 560–620 nm emission filter. Images were acquired with objective lenses ranging from × 20 to × 60 (isolated tube configuration) at a resolution of 1024 × 1024 pixels and an aspect ratio of 1:1.

### 2.2 Electronic Absorption Spectroscopy in the UV–Vis and Determination of Optical Band Gap Energies

The absorption and diffuse reflectance spectra of TiNts, TiNts/Eu, and TiNts­[Eu­(tta)_3_phen] were recorded using a Shimadzu UV–vis Spectrophotometer model 2600i with ISR-2600 Plus integrating sphere. Samples were prepared from small amounts of the solid materials, and measured at room temperature in a powder sample holder support. Spectra were collected over the wavelength range of 220–1400 nm, with a scan rate of 400 nm min^–1^. The resulting data were used to estimate the optical band gap energies of the materials following the methodologies of Tauc^36^ and Davis and Mott.^37^ This approach is based on the calculation of the absorption coefficient (α), expressed in eq 2:

2 $${\left(ahv\right)}^{1/y}=\left(hv-E_{g}\right)$$

In eq 2, α is the absorption coefficient, *h* is Planck’s constant (6.63 × 10^–34^ J s), ν is the radiation frequency, *E*_*g*_ is the band gap energy, *y* is a parameter that depends on the type of electronic transition. For direct and indirect allowed transitions, *y* takes the values 1/2 and 2, respectively.^36,37^ In this work, indirect transitions were considered due to the clearer spectral visualization. Optical band gap energies were determined from eq 2 by extrapolating the linear portion of the Tauc plot.^36^ Additionally, *E*_*g*_ can be calculated from the wavelength using eqs 3 and 4, where *c* is the velocity of light in vacuum 2.998 × 10^8^ m s^–1^.

3 $$E_{g}=\frac{hc}{\lambda }$$

4 $$E_{g}=\frac{1240}{\lambda }$$

### 2.3 Titanate Nanotubes (TiNts) Synthesis

The TiNts were synthesized via an alkaline hydrothermal method using commercial TiO_2_ powder as the precursor.^14^ In a sealed Teflon reactor, 1.0 g of TiO_2_ was mixed with 100 mL of NaOH (10 mol L^–1^), and heated at 120 °C for 24 h. After cooling, the resulting white precipitate was collected and washed with HCl (0.1 mol L^–1^) until pH dropped below 3.0, followed by washing with deionized water until neutral pH was reached. The solid was then dried under vacuum at 50 °C for 24 h to obtain a white powder in 89% yield. The material was characterized by XRD, TEM, SEM, and UV–vis spectroscopy.

### 2.4 Adsorption of Europium Ions onto Titanate Nanotubes (TiNts/Eu)

The TiNts/Eu hybrid was synthesized by dispersing 100 mg of TiNts in 30 mL of a 1% sodium dodecyl sulfate (SDS) solution in 95% ethanol in a beaker, followed by ultrasonication for 3 h. After removing the excess supernatant, then 10 mL of ethanol and 10 mL of a 0.5 mmol Eu­(NO_3_)_3_ solution were added to the material. The mixture was sonicated for 1 h. The resulting white solid (TiNts/Eu) was washed with water and ethanol, dried overnight at 60 °C, and characterized by FTIR, TEM, SEM/EDS, UV–vis spectroscopy, confocal microscopy, photoluminescence spectroscopy, and antibacterial assays.

### 2.5 Coordination of Eu^3+^ Ions to TiNts, tta, and phen: Synthesis of TiNts­[Eu­(tta)_3_phen]

The TiNts­[Eu­(tta)_3_phen] nanohybrid was synthesized using a molar ratio of 1:3:1 between Eu­(NO_3_)_3_, 0.5 mmol, 10 mL, tta (1.5 mmol, 333.30 mg), and phen (0.5 mmol, 90.10 mg). First, 100 mg TiNts were dispersed in 30 mL of a 1% SDS solution in 95% ethanol and sonicated for 3 h. After removing the supernatant, 10 mL of ethanol and 10 mL of 0.5 mmol Eu­(NO_3_)_3_ solution were added to the remaining solid, and the mixture was sonicated for 1 h. Separately, tta (330.30 mg) and phen (90.10 mg) were dissolved in 30 mL of ethanol, and the pH was adjusted to 6.5–7.0 using 0.1 mol L^–1^ NaOH. This solution was stirred for 1 h and then slowly added to the europium-functionalized nanotubes (TiNts/Eu). The mixture was maintained at room temperature for 12 h, and the resulting reddish solid TiNts­[Eu­(tta)_3_phen] was washed with water and ethanol and dried overnight at 60 °C. The final material was characterized by the same techniques used for the TiNts/Eu sample.

### 2.6 Antibacterial Activity Assays

The antimicrobial activity of TiNts, tta, phen, [Eu­(tta)_3_phen], TiNts/Eu, and TiNts­[Eu­(tta)_3_phen] was evaluated against *E. coli* ATCC 25922 and *S. aureus* ATCC 25923 using two different methods following the general recommendations of the CLSI guidelines.^38,39^

For the agar well diffusion method, Mueller–Hinton agar plates were inoculated with bacterial suspensions previously adjusted to a 0.5 McFarland standard (∼1.5 × 10^8^ CFU mL^–1^).^38^ Wells of 6 mm in diameter were aseptically perforated in the solidified agar, and 50 μL of each test solution were added into the wells. The samples were tested at concentrations of 3.0, 6.0, 12.0, 25.0, 50.0, and 100 mg mL^–1^. Sterilized deionized water served as the negative control, and gentamicin (10 μg) was used as the positive control. The plates were incubated at 35 °C for 24 h under dark conditions, and the antimicrobial effect was determined by measuring the diameter of the inhibition zones formed around each well. All experiments were conducted in triplicate.

MIC assays were performed in a 96-well microtitration plate using the broth microdilution method.^39^ Stock solutions of the TiNts­[Eu­(tta)_3_phen] was prepared at 100 mg mL^–1^ and 50 μL of each stock solution was added to the first well containing 50 μL of Mueller–Hinton broth. Serial 2-fold dilutions were then made by transferring 50 μL from each well to the next, generating a gradient of decreasing concentrations across the plate. Subsequently, 50 μL of a bacterial suspension (∼1 × 10^6^ CFU mL^–1^), obtained from a 0.5 McFarland standard, was added to each well, yielding a final volume of 150 μL per well, and final TiNts­[Eu­(tta)_3_phen] concentrations from 33.3 to 0.130 mg mL^–1^. Positive and negative controls were included according to CLSI recommendations. The plates were incubated at 35 °C for 24 h, and bacterial growth was visually assessed to determine the MIC, defined as the lowest concentration of antimicrobial agent that completely inhibited visible bacterial growth in the wells, as observed by the unaided eye.

## 3 Results and Discussion

### 3.1 Nanohybrid TiNts/Eu and TiNts­[Eu­(tta)_3_phen]

The successful synthesis of TiNts, TiNts/Eu, and TiNts­[Eu­(tta)_3_phen] was confirmed through a combination of analytical techniques.

#### XRD Analysis

The XRD patterns of TiNts display characteristic peaks of multiwalled titanate nanotubes at 2θ = 10, 24.5, 28, and 48, corresponding to the crystallographic planes (200), (110), (211), and (020), respectively (Figure S1). These reflections indicate the coexistence of anatase and rutile phases, as well as orthorhombic hydrogen titanates, such as H_2_Ti_2_O_4_(OH)_2_ or Na_2_Ti_3_O_7_.^40–42^ SEM and TEM analyses further confirm the formation of TiNts, revealing tubular morphology and multiwalled titanate nanotubes structures (Figures S2 and, S3a) with diameters of approximately 7.0 nm (Figure S3b).

#### FTIR Analysis

FTIR spectroscopy was also used to investigate the formation of the nanomaterials (Figure 1). The spectrum of TiNts reveals bands associated with water and hydroxyl groups, including the H–O–H bending vibration at 1621 cm^–1^ and a strong O–H stretching vibration at 3360 cm^–1^. The band at 896 cm^–1^ is attributed to lattice vibrations of Ti–O and Ti–O–Ti bonds, confirming the presence of the TiO_6_ octahedral framework.^42,43^ Adsorption of Eu^3+^ ions into TiNts via ion exchange is evidenced by the replacement of interlayer Na^+^ ions. The disappearance of the Ti–O–Na band at 896 cm^–1^, as reported by Wu *et al*.,^43^ further confirms this substitution. Additionally, a weak band at 540 cm^–1^, attributed to Eu–O stretching arising from interactions between TiNts and europium ions, appears as suggested by Lv *et al*.^44^ and Juan *et al*.^45^ The band near 1400 cm^–1^ corresponding to S = O stretching, along with the strong bands at near 1500 cm^–1^ and 2922 cm^–1^ attributed to C–H symmetric and asymmetric stretching vibrations, respectively, confirm the presence of SDS.^46^ The formation of the TiNts­[Eu­(tta)_3_phen] complex, in turn, is further evidenced by axial distortion of the carbonyl groups, observed as bands around 1624 cm^–1^. Symmetrical axial vibrations of the unsaturated C = C bonds appear at lower wavenumbers, within the 1585–1535 cm^–1^ region. Stretching bands associated with the phen ligand, particularly antisymmetric C–N stretching vibrations, were detected between 1601 and 1614 cm^–1^, overlapping with the carbonyl bands of the tta ligand and suggesting possible interactions. Bands in the 724–843 cm^–1^ range are attributed to angular symmetric C–H vibrations, resulting from the coordination of phen with Eu^3+^ ions. Coordination between TiNts and Eu^3+^ ions is further supported by shifts observed near 500 cm^–1^, as reported by Balasanthiran^47^ and Dandekar *et al*.^48^

**Figure 1:**
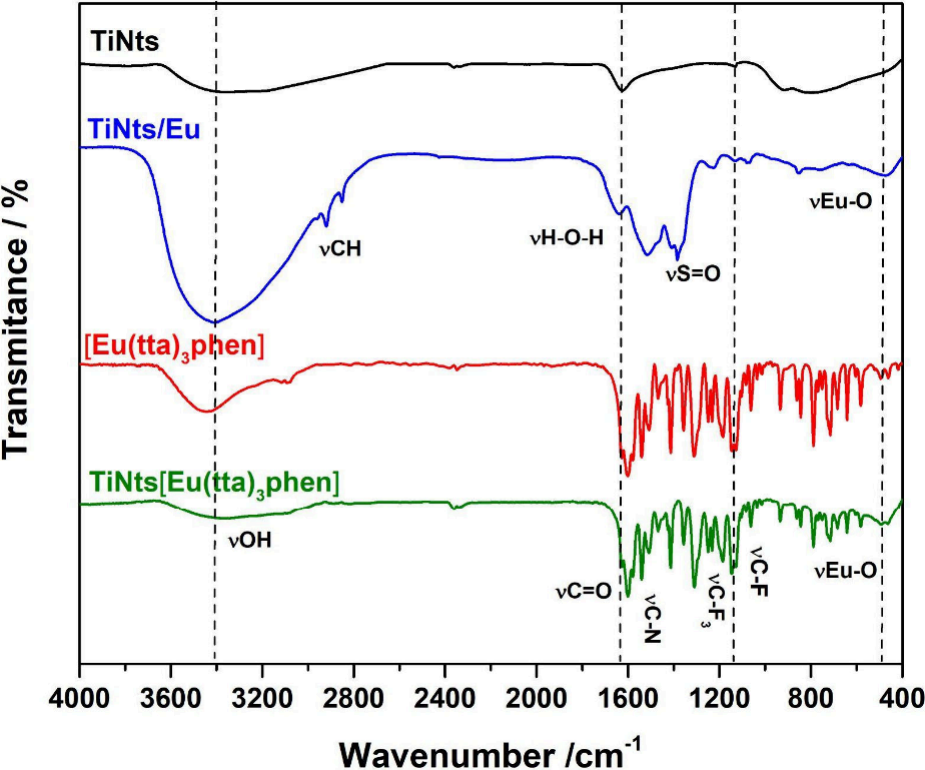
FTIR spectra of TiNts, TiNts/Eu, [Eu­(tta)_3_phen], and TiNts­[Eu­(tta)_3_phen] samples.

#### SEM and TEM-Mapping and Fluorescence Confocal Microscopy Analysis

The size distribution, surface morphology, and optical properties of freshly prepared TiNts/Eu and TiNts­[Eu­(tta)_3_phen] materials were examined by SEM, TEM, and CLSM (Figure 2). SEM (Figure 2a,e) and TEM (Figure 2b,f), in TiNts/Eu exhibits a nonuniform, dense morphology characterized by clusters of tubular structures corresponding to titanate nanotubes (Figure 2a). In contrast, the luminescent hybrid TiNts­[Eu­(tta)_3_phen] displays a notably different morphology, with rectangular structures of varying lengths and widths, which, according to Song *et al*.,^49^ can be attributed to the [Eu­(tta)_3_phen] complex. The appearance of these cube-like structures suggests interactions between the complex and the TiNts surface, supporting the successful formation of the TiNts­[Eu­(tta)_3_phen] nanohybrid.

**Figure 2:**
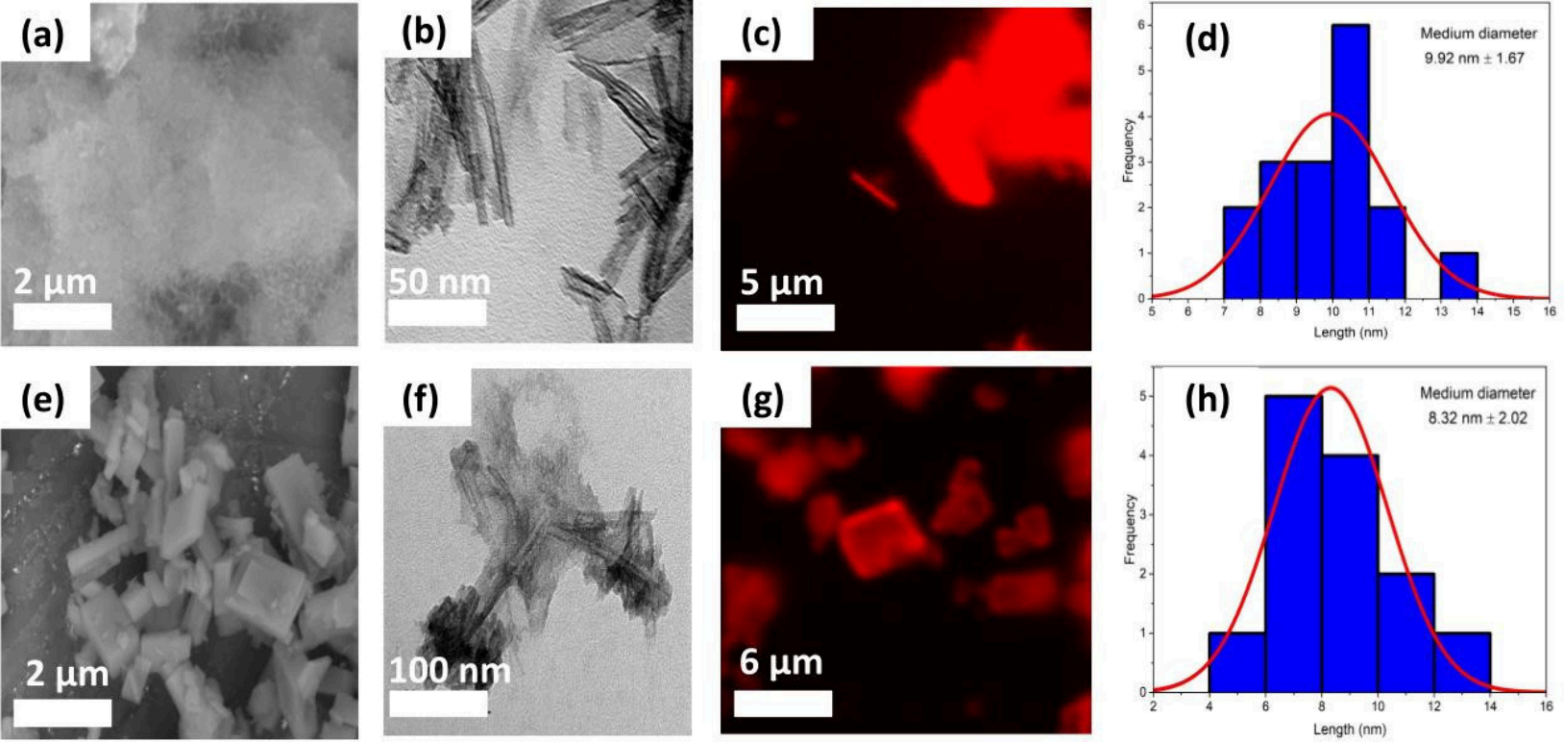
(a–d) SEM, TEM, fluorescence confocal images λ_ex_ = 405 nm, analysis at 560–620 nm range and histogram along with the outer diameter distribution histogram of the TiNts/Eu sample. (e–h) Analogous data for the TiNts­[Eu­(tta)_3_phen] nanohybrid.

Confocal microscopy images of TiNts/Eu and TiNts­[Eu­(tta)_3_phen] (Figure 2c,g) show that both samples emit red luminescence (560–620 nm) upon laser excitation. The images confirm the presence of TiNts in both hybrid materials. Complementary CLSM studies (λ_ex_ = 405 nm) demonstrate the optical activity of the hybrid, revealing characteristic Eu^3+^ emission at 615 nm corresponding to the ^5^D_0_ → ^7^F_2_ transition.

Additionally, the TiNts/Eu and TiNts­[Eu­(tta)_3_phen] complex and luminescent hybrids composition was further examined by EDS analysis (Table 1).

**Table 1:** EDS Data of TiNts, TiNts/Eu, [Eu­(tta)_3_phen], and TiNts­[Eu­(tta)_3_phen]

**Atom**	**TiNts/**at. %	**TiNts/Eu/**at. %	**[Eu(tta)_3_phen]/**at. %		****TiNts**[Eu(tta)_3_phen]/**at. %	
**C**			46.6 ± 4.6	49.2 ± 2.3	50.9 ± 5.08	53.3 ± 0.6
**O**	56.4 ± 4.0	45.0 ± 3.5	11.5 ± 2.6	14.0 ± 2.6	12.5 ± 3.2	13.8 ± 3.1
**Ti**	37.2 ± 2.8	22.4 ± 3.2			5.0 ± 6.6	
**Na**	6.4 ± 0.2	4.7 ± 0.1			0.53 ± 0	
**F**			22.3 ± 2.4	28.0 ± 1.7	19.0 ± 2.6	23.2 ± 3.1
**S**		1.1 ± 0.2	5.99 ± 0.3	6.02 ± 0.3	6.3 ± 2.3	7.0 ± 2.2
**N**				2.3 ± 2.3		4.5 ± 2.3
Eu		19.1 ± 1.6	12.7 ± 5.2		9.5 ± 3.1	

Due to instrumental limitations, it was not possible to simultaneously determine the elemental composition of titanium (Ti), europium (Eu), and nitrogen (N). Therefore, two separate EDS analyses were conducted for the complex [Eu­(tta)_3_phen], and for the hybrid material TiNts­[Eu­(tta)_3_phen]. The first analysis targeted nitrogen, while the second focused on titanium and europium. Elemental analysis of TiNts revealed predominant amounts of oxygen (56.4%) and titanium (37.2%), consistent with the expected titanate nanotube structure [H_2_Ti_2_O_4_(OH)_2_ or Na_2_Ti_3_O_7_]. Elemental analysis of the TiNts/Eu material, functionalized with SDS, revealed the presence of sulfur (1.1%), sodium (4.7%) and europium at 19.1%. For the [Eu­(tta)_3_phen] complex, carbon, oxygen, fluorine, sulfur, and nitrogen were detected, consistent with the tta and phen ligands, alongside europium at 12.7%. Similarly, in the TiNts­[Eu­(tta)_3_phen] hybrid material, the presence of these elements associated with the ligands was also confirmed. Additionally, europium (9.5%) and titanium (5.0%) were detected, confirming the effective incorporation of both [Eu­(tta)_3_phen] and TiNts into the nanohybrid. These findings collectively validate the synthesis of the nanohybrid materials.^49–52^

#### Optical and Electronic Properties

The optical properties of TiNts, TiNts/Eu, [Eu­(tta)_3_phen], and TiNts­[Eu­(tta)_3_phen], in the powder form, were investigated using UV–Vis absorption spectroscopy and photoluminescence (PL) (Figure 3). The spectra of TiNts and TiNts/Eu exhibit strong absorption bands in the UV region (below 300 nm), attributed to the excitation of the electron O 2p orbitals in the valence band to Ti 3d levels in the conduction band or Eu *4f* states.^53–55^

**Figure 3:**
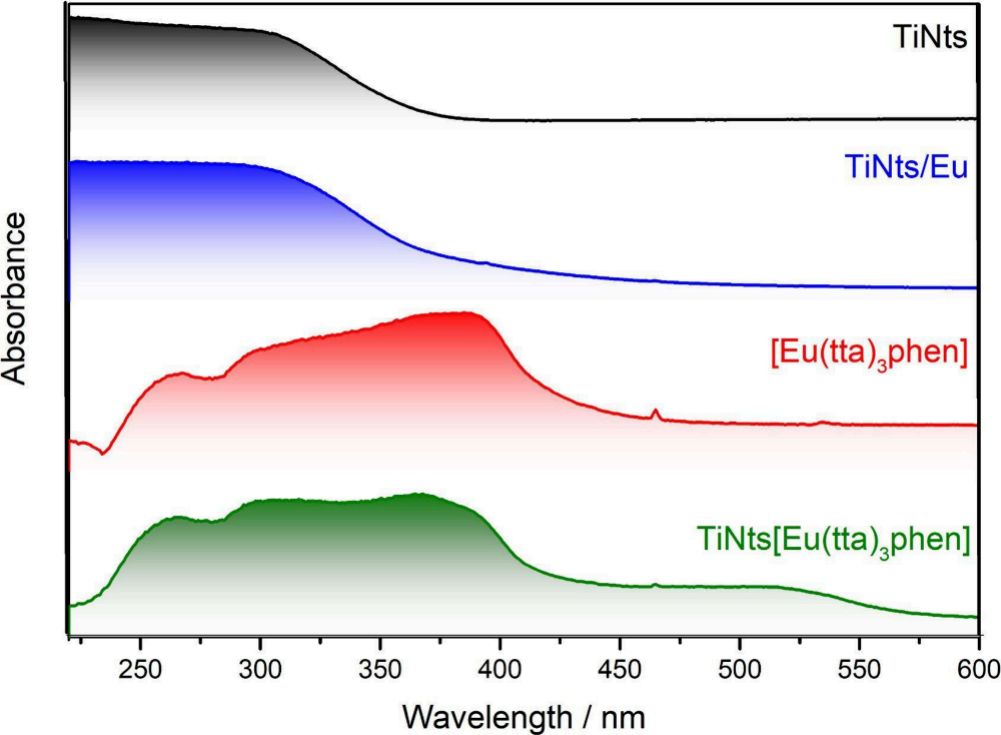
UV–vis spectra of TiNts, TiNts/Eu, [Eu­(tta)_3_phen], and TiNts­[Eu­(tta)_3_phen] in solid form.

Upon modification of TiNts with Eu^3+^ ions and the ligands tta and phen, additional absorption bands appear around 400 nm. The phen ligand displays absorption bands in the 230–260 nm range, corresponding to π → π* and n → π* transitions, while tta exhibits bands at 250 and 330 nm, characteristic of π → π* transitions of the thiophene ring and n → π* transitions of the C=O group.^56^ These modifications enable the hybrid materials to absorb light in the visible region. The observed absorption features are attributed to the introduction of Eu *4f* orbitals within the band gap and the subsequent charge-transfer transition between the f-electrons of the dopant and the conduction band of TiNts.

As shown in Figure 4, the optical band gap energies of TiNts, TiNts/Eu, and TiNts­[Eu­(tta)_3_phen], determined using the Tauc relation (eq 2),^36^ are 3.37, 2.72, and 3.23 eV, respectively. The reduction in band gap upon the europium­(Eu^3+^) indicates enhanced light absorption and improved photocatalytic potential. This narrowing is likely due to interaction between Eu^3+^ ions and the titanate lattice, which may lead to the formation of intermediate energy states within the band gap or the creation of impurity levels.^57^

**Figure 4:**
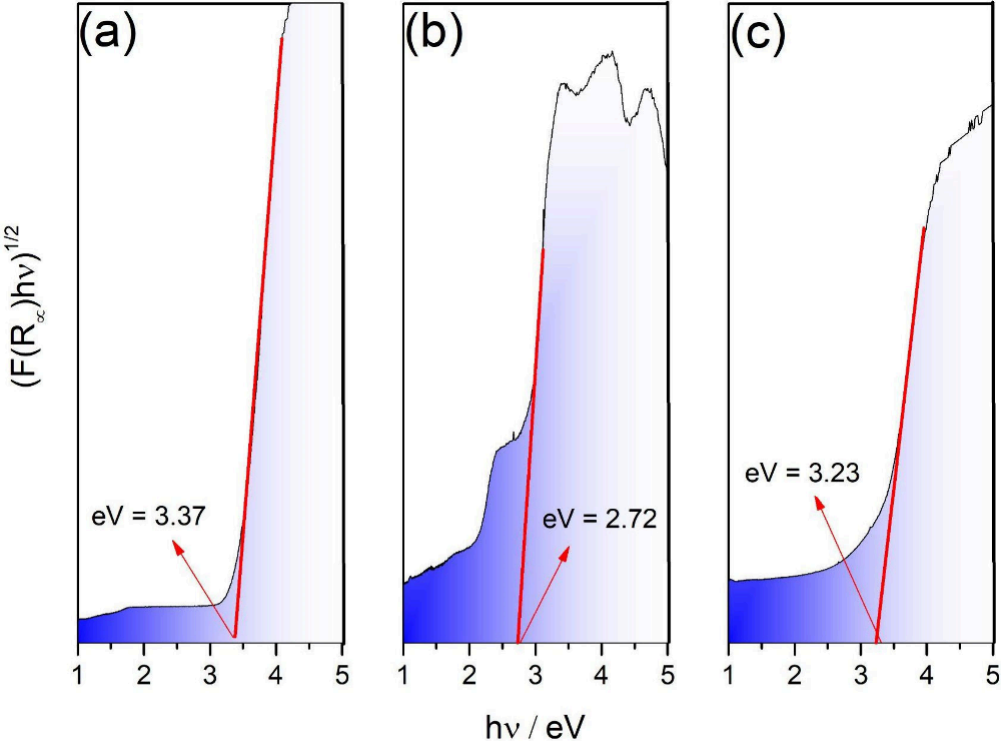
Optical band gap evaluation for (a) TiNts, (b) TiNts/Eu, and (c) TiNts­[Eu­(tta)_3_phen] samples.

Doping TiNts with Eu^3+^ results in a notable decrease in the band gap to 2.72 eV, consistent with the formation of midgap states or defect levels induced by the lanthanide dopant. However, in the hybrid system TiNts­[Eu­(tta)_3_phen], the band gap increases to 3.23 eV compared to the Eu-doped TiNts. This increase is likely due to the suppression or passivation of low-energy defect states by the coordinating tta and phen ligands, which absorb strongly in the ultraviolet region (approximately 270 and 350 nm, respectively). These ligands act as “antenna”, modifying the local electronic environment of the Eu^3+^ ion and influencing the energy structure of the hybrid system.^58^ Additionally, Pode *et al*. reported band gap values of 3.42, 3.41, 3.39, 3.38, and 3.35 eV for the Eu­(tta)_3_phen complex dissolved in chloroform, toluene, tetrahydrofuran (THF), acetic acid, and formic acid, respectively. These findings further demonstrate how the coordination environment and solvent polarity can influence the electronic transitions of such complexes.^59^

Europium (III) ions act as electron acceptors, promoting the formation of a Schottky barrier that enhances the separation of photogenerated electron–hole pairs, thereby improving charge carrier dynamics and quantum efficiency. Consequently, the hybrid materials TiNts/Eu and TiNts­[Eu­(tta)_3_phen] exhibit enhanced photoactivity, highlighting their potential for applications in photocatalysis and antimicrobial treatments.^60,61^

These findings are consistent with previous reports. Liao *et al*.^62^ observed similar band gap shifts upon lanthanide doping, while Capizzi *et al*.^63^ attributed intermediate energy levels to structural defects, such as oxygen vacancies, typically associated with [TiO_5_] and [TiO_6_] coordination environments. Additionally, Baccaro *et al*.^64^ and Diamandescu *et al*.^65^ reported band gap values in the 2.9–3.0 eV range for europium-modified titanates, in agreement with the present results.

#### Photoluminescence Spectral Analysis

Comparison of the excitation spectra of TiNts/Eu and TiNts­[Eu­(tta)_3_phen] reveals distinct optical features (Figure 5a,b). NtsTi/Eu exhibit well-defined intraconfigurational transitions of Eu^3+^ (e.g., ^7^F_0_ → ^5^L_6_, ^7^F_1_ → ^5^D_3_) as well as ligand-to-metal charge transfer bands, indicating that Eu^3+^ occupies noncentrosymmetric sites. In contrast, TiNts­[Eu­(tta)_3_phen] display a broad excitation band from 250 to 450 nm, arising from strong absorption tta and phen ligands. This absorption overlaps the Eu^3+^ transitions (antenna effect), enhancing overall while masking individual *f*–*f* transitions.

**Figure 5:**
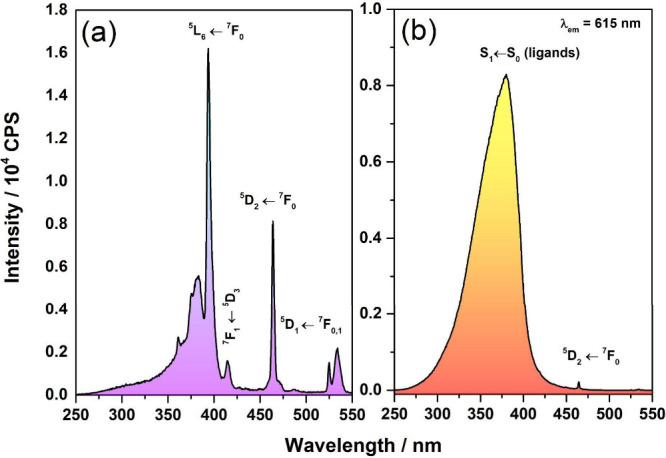
Excitation spectra at λ_em_ = 615 nm of (a) TiNts/Eu and (b) TiNts­[Eu­(tta)_3_phen] powders at room temperature.

The emission spectra of TiNts­[Eu­(tta)_3_phen] were recorded at the excitation wavelengths of λ_ex_ = 377, 393, and 464 nm (Figure 6b). The spectra show maximum emission intensity at the hypersensitive ^5^D_0_ → ^7^F_2_ transition. Additionally, otherwise forbidden transitions, ^5^D_0_ → ^7^F_0_ and ^5^D_0_ → ^7^F_3_ are observed at 580 and 655 nm, respectively, due to the relaxation of selection rules in the low-symmetry environment surrounding the Eu^3+^ ion.^66^ The emission spectra profiles confirm efficient energy transfer from the titanate matrix and the coordinated ligands (tta and phen) to the Eu^3+^ center. As expected, the ligands function as antennas, absorbing excitation energy and sensitizing the europium ion via the antenna effect, the photophysical performance is strongly influenced by the choice of coordinating ligands, phen and tta were strategically selected for their ability to act as efficient antennas, transferring energy from their triplet states to the Eu^3+^ ^5^D_1_ level. The energy alignment of both ligands with Eu^3+^ enables highly efficient sensitization, while their structural features such as rigidity and low-vibrational CF_3_ groups suppress nonradiative losses.^67^

**Figure 6:**
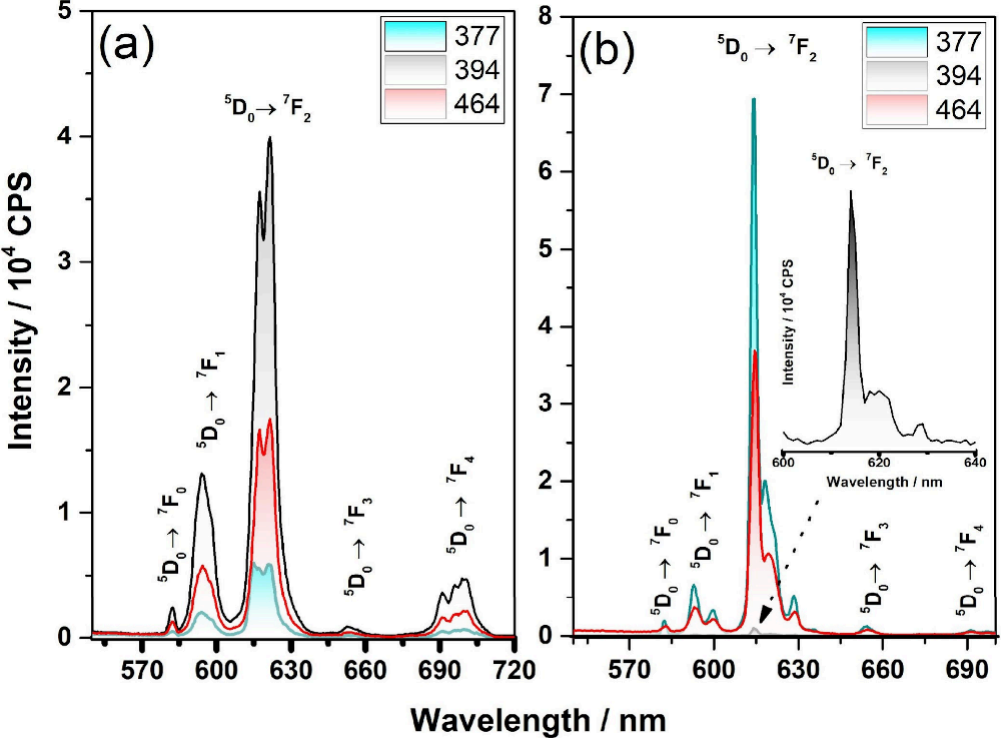
Emission spectra at λ_ex_ = 377, 391, and 461 nm of the (a) TiNts/Eu and (b) TiNts­[Eu­(tta)_3_phen] powders at room temperature.

In comparison, the emission spectra of TiNts/Eu (Figure 6a), also fixing excitation at different wavelengths, exhibit broader and less-resolved bands than those of TiNts­[Eu­(tta)_3_phen] (Figure 6b), indicating less selective energy transfer and greater overlap of electronic transitions. Nevertheless, the characteristic 4*f*–4*f* transitions of Eu^3+^, specifically ^5^D_0_ → ^7^F*_n_* (*n* = 0, 1, 2, 3, 4), remain discernible. Among these, the ^5^D_0_ → ^7^F_2_ forced electric dipole transition, observed in the 616–622 nm range, is dominant, confirming that Eu^3+^ ions occupy noncentrosymmetric coordination environments, which enhances luminescence intensity. The synergistic action of phen and tta significantly enhances the antenna effect, resulting in improved luminescence intensity and stability in the TiNts­[Eu­(tta)_3_phen] hybrid. This optimized energy transfer also contributes to enhanced biological performance, suggesting a strong correlation between ligand-assisted sensitization and the material’s multifunctional behavior.^68^

#### Excited-State Lifetime Analysis

Table 2 summarizes the lifetimes and pre-exponential factors of TiNtsEu, TiNts­[Eu­(tta)_3_phen], and [Eu­(tta)_3_phen], determined from biexponential fits of the luminescence decay curves.

**Table 2:** Values of Excitation (λ_ex_) and Emission (λ_em_) Wavelengths, Pre-exponential Factors (*A*_1_ and *A*_2_), Associated Lifetimes (τ_1_ and τ_2_), and Overall Lifetimes (τ_overall_) Obtained from Biexponential Fitting of the Luminescence Decay Curves of TiNts/Eu, TiNts­[Eu­(tta)_3_phen] and [Eu­(tta)_3_phen]

**Sample**	**λ_ex_/nm**	**λ_ex_/nm**	***A*_1_**	***A*_2_**	**τ_1_/ms**	**τ_2_/ms**	**τ_overall_/ms**
**TiNts/Eu**	377.5	614	0.74	0.43	0.23	0.76	0.58
	394.0		0.68	0.51	0.20	0.52	0.42
	464.0		0.88	0.33	0.21	0.57	0.39
**TiNts[Eu(tta)**_**3**_ **phen]**	377.5	614	0.14	0.93	0.27	0.87	0.84
	464.0		0.74	0.32	0.63	1.18	0.87
**[Eu(tta)**_**3**_ **phen]**	377.5	614	0.09	0.98	0.30	0.81	0.79
	464.0		0.06	1.01	0.17	0.77	0.76

The obtained lifetimes fall within the typical range reported for Eu^3+^-based compounds (0.2 to 2.0 ms), depending on the chemical environment and the nature of the coordinating ligands. The presence of two decay components (Figure 6a,b) indicates the existence of two distinct Eu^3+^ coordination environments, likely arising from variations in local symmetry or different interactions with solvents, ligands, or the titanate nanostructure surface.^69,70^

Furthermore, lifetime measurements under different excitation wavelengths show that excitation at 377 nm produces longer lifetimes than at 394.0 and 464.0 nm. This behavior is likely due to more efficient ligand-to-Eu^3+^ energy transfer, reflecting enhanced sensitization of the lanthanide ion via the antenna effect. It also explains the observed increase in luminescence lifetime upon incorporation of the ligands into the Eu^3+^ coordination sphere.^71^

Finally, the [Eu­(tta)_3_phen] complex, used as a reference, exhibits a monoexponential luminescence decay, reflecting its high structural symmetry and homogeneity. Upon immobilization on the surface of the titanate nanostructure surface, the decay profile shifts to a well-defined biexponential behavior, as expected due to the broader range of interactions between the complex and the titanate surface.^72^ Luminescence decay curves of TiNts/Eu, TiNts­[Eu­(tta)_3_phen] are shown in Figure Supporting Information (Figures S4 and S5).

#### Photostability Evaluation

The photostability of the TiNts/Eu, TiNts­[Eu­(tta)_3_phen] and [Eu­(tta)_3_phen] was evaluated (Figure S6).

During the first stages of continuous UV exposure, the TiNts/Eu system exhibited a slight increase in luminescence intensity. This behavior can be attributed to the desorption of water molecules or surface hydroxyl groups coordinated to Eu^3+^ ions and on the TiNts surface.^73^ These adsorbed species can act as nonradiative quenching centers, dissipating energy that would otherwise contribute to radiative emission. Upon UV irradiation, the desorption or photoreduction of these molecules decreases the density of quenching centers, allowing more charge carriers to recombine radiatively and resulting in a gradual increase in luminescence intensity.

In contrast, in organic or hybrid organic–inorganic systems, continuous exposure to high-energy radiation, such as the UV light employed in this study, can more easily promote degradation and/or irreversible photoreduction processes that lead to intensity suppression.^74,75^ This trend is clearly observed for the [Eu­(tta)_3_phen] complex and for the hybrid material, both of which exhibited a gradual decrease in emission intensity over time. However, it is also evident that the incorporation of the complex onto the TiNts surface results in a small but noticeable enhancement in the overall stability of the system. Finally, the luminescence suppression observed for the hybrid system remained below 10% after 1 h of continuous UV exposure, indicating that the TiNts-based hybrid exhibits good stability and robustness under UV irradiation.

### 3.2 Antibacterial Activity Assays

The antibacterial properties of TiNts, TiNts/Eu, tta, phen, [Eu­(tta)_3_phen], and TiNts­[Eu­(tta)_3_phen] was first evaluated by the agar well diffusion method against *Escherichia coli* ATCC 25922 and *Staphylococcus aureus* ATCC 25923. The corresponding results are summarized in Table 3, and the inhibition halos are illustrated in Figure 7.

**Table 3:** Antibacterial Activity of the Nanomaterials against *S. aureus* ATCC 25923 and *E. coli* ATCC 25922^a^

	***S. aureus***					***E. coli***				
	**Concentration (mg mL^–1^)**					**Concentration (mg mL^–1^)**				
**Material**	**3**	**6**	**12**	**25**	**Control**	**3**	**6**	**12**	**25**	**Control**
**tta**										
**Phen**	10.0 ± 0.2	13.0 ± 1.7	20.5 ± 3.5			13 ± 3.0	18.5 ± 1.5	20.0 ± 1.0		
**[Eu(tta)**_**3**_ **phen]**					12					10
**TiNts**					13					7
**TiNts/Eu**				5.0 ± 0	12		7.0 ± 0	9.5 ± 0.5	11 ± 0	16
**TiNts[Eu(tta)**_**3**_ **phen]**			7.0 ± 3.0	12.5 ± 2.5	14			10 ± 4.0	12 ± 1	15

a ***Note:*** Control - corresponds to the gentamicin (10 μg). All tests were performed in triplicate for each bacterium. The zones of inhibition were expressed in millimeters (mm).

**Figure 7:**
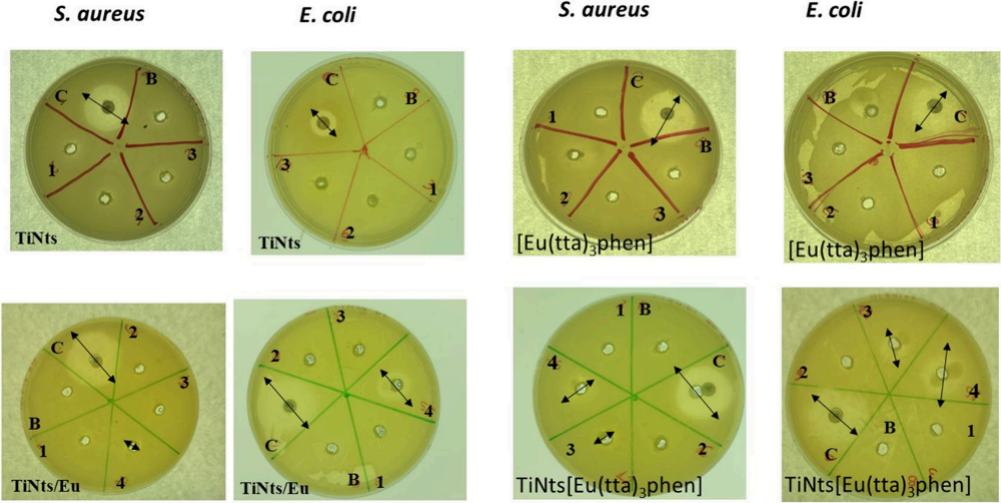
Antibacterial activity of TiNts, [Eu­(tta)_3_phen], TiNtsTi/Eu and TiNts­[Eu­(tta)_3_phen] against *S. aureus* ATCC 25923 *and E. coli* ATCC 25922. ***Note:*** 1 (3 mg mL^–1^), 2 (6 mg mL^–1^), 3 (12 mg mL^–1^), 4 (25 mg mL^–1^). B, corresponds to the negative control (sterilized deionized water); C, corresponds to the gentamicin (10 μg). All tests were performed in triplicate for each bacterium.

The phen ligand showed measurable antibacterial effects, with inhibition zones of 10–20 mm against *S. aureus* and 13–20 mm against *E. coli*. While, tta did not exhibit significant activity at any tested concentration, with no relevant inhibition zones observed. These findings suggest that phen contributes to the antimicrobial properties of the corresponding metal complexes, whereas tta appears to play a minimal role (Figure S7).

In contrast, both TiNts/Eu and TiNts­[Eu­(tta)_3_phen] displayed clear antibacterial activity (Figure 7). *S. aureus* and *E. coli* were sensitive to both hybrid materials, with inhibition zones ranging from 5 to 12.5 mm for *S. aureus* and from 7 to 12 mm for *E. coli*. Notably, *E. coli* demonstrated greater susceptibility compared to *S. aureus*.^76^ An additional study was performed by varying the material concentration to 50 and 100 mg. The corresponding results are depicted in Figure 8.

**Figure 8:**
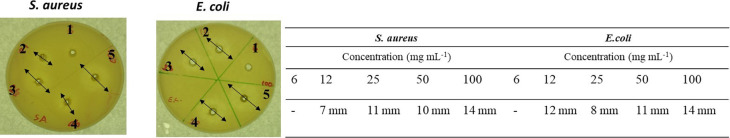
Antibacterial activity of TiNts­[Eu­(tta)_3_phen] against *S. aureus* ATCC 25923 and *E. coli* ATCC 25922. ***Note:*** 1 (6 mg mL^–1^), 2 (12 mg mL^–1^), 3 (25 mg mL^–1^), 4 (50 mg mL^–1^), 5 (100 mg mL^–1^).

The 100 mg concentration showed inhibition near 14 mm, indicating significant antimicrobial activity. These results are very similar to those obtained with gentamicin, which showed inhibition zones of 13 ± 3 mm. This similarity suggests that, at the tested concentration, the compound exhibits comparable efficacy to gentamicin, a widely used reference antibiotic in susceptibility tests.

Additionally, a specific MIC study was conducted for the TiNts­[Eu­(tta)_3_phen] hybrid, with results showing MIC values of 0.26 mg mL^–1^ against *E. coli* and 0.52 mg mL^–1^ against *S. aureus*. Therefore, it can be observed that the hybrid exhibited greater efficacy against the Gram-negative bacterium. The TiNts­[Eu­(tta)_3_phen] hybrid demonstrated relevant antimicrobial activity, confirmed by multiple experimental approaches. Agar diffusion assays showed inhibition zones ranging from 7 to 14 mm for *S. aureus* and from 8 to 14 mm for *E. coli*, considering concentrations between 12 and 100 mg mL^–1^. These results confirm the efficacy of the TiNts­[Eu­(tta)_3_phen] hybrid, especially at higher concentrations, highlighting its potential as an alternative antimicrobial agent with performance comparable to commercial antibiotics.

Chloramphenicol and vancomycin are widely used antibiotics for the treatment of bacterial infections.^77^ The susceptibility of *S. aureus* and *E. coli* to these agents depends on both the antibiotic concentration and the bacterial strain.^6,78^ According to tests reported by Cruz *et al*.^14^ *E. coli* and *S. aureus* are sensitive to chloramphenicol, exhibiting inhibition zones of 20 mm and 14 mm, respectively. In contrast, both bacteria display resistance to vancomycin, with inhibition zones smaller than 8 mm.

Although metal oxide-based nanomaterials are known to exhibit intrinsic antimicrobial activity against both Gram-positive and Gram-negative bacteria due to their nanoscale properties, the enhanced antibacterial performance observed in this study is attributed to the synergistic interaction between TiNts, tta, phen and Eu^3+^ ions. This cooperative effect increases cytotoxicity toward bacterial cells, leading to stronger inhibition compared with materials lacking surface modification.

The antibacterial activity observed in the europium-functionalized titanate nanotubes may involve multiple mechanisms, including Eu^3+^ induced oxidative stress and ligand mediated membrane disruption. Eu^3^ complexes are well-known for their dual function as antimicrobial agents and luminescent probes for reactive oxygen species (ROS), particularly in biological systems. Previous studies have demonstrated their utility in monitoring cellular oxidative stress. Xiao *et al*. demonstrated a ratiometric luminescence probe for highly reactive oxygen species based on lanthanide complexes, enabling real-time monitoring of ROS in cells.^79^ Galaup *et al*. highlight luminescent lanthanide complexes as promising tools for ROS biosensing and for understanding oxidative mechanisms and metal–Aβ interactions that drive Alzheimer’s disease.^80^ Karami *et al*. showed that lanthanide-doped materials can enhance ROS generation under NIR light, achieving over 99.9% bacterial kill rates via synergistic release of silver ions and ROS in photodynamic therapy.^81,82^

Despite this supporting evidence, the response of bacterial cells to oxidative stress is multifactorial and often species-specific. Here, the enhanced antibacterial performance and luminescent properties of the TiNts­[Eu­(tta)_3_phen] hybrid, compared to the TiNts/Eu material, suggest that coordination with organic ligands may play a role in modulating both photophysical and biological activity.

A comparative analysis between previously reported antibacterial nanohybrid and the synthesized TiNts/Eu and TiNts­[Eu­(tta)_3_phen] is shown in Table 4.

**Table 4:** Comparison of the Antibacterial Performance of the Synthesized Nanohybrid with Data Reported in the Literature

**Material**	***S. aureus*/mm**	***E. coli*/mm**	**Ref**
Eu^3+^ induced polyelectrolyte nanoaggregates (EIPAs)	29.5	-	Wang *et al*.^83^
[Eu(phen)_2_(OH_2_)_2_(Cl)_2_](Cl)(H_2_O)	31.6	-	Alfi *et al*.^84^
TiO_2_ NPs	12.3–12.9	9.7–10.9	Khashan *et al*.^85^
Eu-doped CeO_2_ nanoparticles	20–24	30–32	Gnanam *et al.* ^86^
Eu:WO_2_ NPs	11–14	-	Subramani and Nagarajan^87^
TiNts/Eu	5	7–11	This study
TiNts[Eu(tta)_3_phen]	7–14	8–14	This study

## 4 Conclusion

Titanate nanotubes (TiNts) were successfully synthesized via an alkaline hydrothermal method, yielding nanostructures with well-defined morphology and moderate crystallinity, as confirmed by TEM and XRD analyses. While pristine TiNts exhibited no antibacterial activity, functionalization with Eu^3+^ ions (TiNts/Eu) significantly enhanced their performance. Moreover, incorporation of the [Eu­(tta)_3_phen] complex (TiNts­[Eu­(tta)_3_phen] imparted strong red luminescence and antibacterial effects against *S. aureus* and *E. coli* arising from the synergistic effects of Eu^3+^, tta, phen and TiNts. These results highlight the potential of lanthanide-doped titanate nanotube hybrids as multifunctional nanomaterials for antimicrobial coatings, sensors, and bioimaging applications.

## Supplementary Material



## Notes

Synthesis of the [Eu­(tta)_3_phen] complex; XRD analysis of TiNts (Figure S1); SEM and TEM images of TiNts (Figures S2 and S3); normalized intensity decay curves of TiNts/Eu as a function of excitation wavelength with inset showing Log_10_ of the curves (Figure S4); normalized intensity decay curves of TiNts­[Eu­(tta)_3_phen] as a function of excitation wavelength with inset showing Log_10_ of the curves (Figure S5); photostability of the TiNts/Eu, TiNts­[Eu­(tta)_3_phen] (Figure S6); antibacterial activity of tta and phen growth of *S. aureus* and *E. coli* (Figure S7) (PDF)
