# Supplementary material for: Synthesis and Characterization of Luminescent and Antibacterial Europium-Titanate nanotubes
Source: ACS Omega. 2025 Dec 13;10(51):63016–27. doi: 10.1021/acsomega.5c08930 (PMC12756813; doi:10.1021/acsomega.5c08930)
Supplement: Supplementary file 1 [file ao5c08930_si_001.pdf]

Supplementary Material

Synthesis and Characterization of Luminescent and Antibacterial Europium-Titanate nanotubes

Enzo O. Borazo[a], Rodolpho A. N. Silva[b], Gabriel L. Colombo[c], Ana M. Pires[c,d], Emilson R. Viana[e], Gustavo H. Couto[a], Renata D. Adati[a], Cristiane Pilissão[a*]

[a] Department of Chemistry and Biology, Federal University of Technology Parana (UTFPR), Curitiba, PR, Brazil.

[b] Department of Sustainable Development and Ecological Transition, University of Eastern Piedmont "A. Avogadro", Vercelli, Italy.

[c] Department of Analytical, Physical-Chemistry and Inorganic Chemistry, Sao Paulo State University (UNESP), Institute of Chemistry, Araraquara, SP, Brazil.

[d] Department of Chemistry and Biochemistry, Sao Paulo State University (UNESP), School of Science and Technology, Presidente Prudente, SP, Brazil.

[e] Department of Physics, Federal University of Technology Parana (UTFPR), Curitiba, PR, Brazil

Corresponding authors: *Cristiane Pilissão (email: pilissao@utfpr.edu.br) and Renata D. Adati (email: renataadati@utfpr.edu.br)

1. Synthesis of the [Eu(tta)$_3$phen] complex

To compare structural changes in the nanotubes, the [Eu(tta)$_3$phen] was prepared and the synthesis carried out by adapting the methodology of Hui-Xia *et al.*[1], using a molar ratio of 1:3:1 for europium (III) (Eu^{3+}, 10 mL, 0.5 mmol), thenoyltrifluoroacetone (tta, 1.5 mmol, 333.30 mg), and 1,10-phenanthroline (phen, 0.5 mmol, 90.10 mg). In a 100 mL beaker, 40 mL of ethanol was mixed with 10 mL of $Eu(NO_3)_3$ 0.5 mmol solution, 330 mg of tta, and 90.10 mg of phen, and the pH was adjusted in the interval of 6.5 to 7.0 adding NaOH aqueous solution (0.1 mol L^{-1}) until the complex precipitation. The mixture was allowed to stand for 12 h, followed by multiple washings with water and ethanol. Finally, the product was dried overnight in an oven at 60 °C, yielding a white solid. After drying, the solid exhibited red emission under a UV lamp of 365 nm.

2. XRD analysis of TiNts

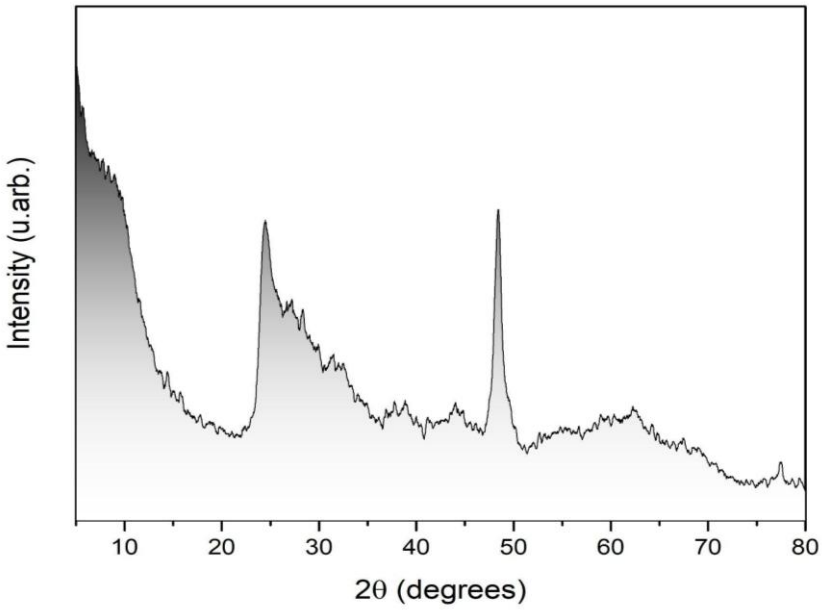

Figure S1. X-ray diffractogram of TiNts.

3. SEM and TEM images of TiNts

The images of SEM and TEM shown in **Figure S2 and S3a, S3b** confirm the formation of TiNts. TEM (**Figure S2 and S3a)** shows that TiNtsi has a tubular morphology and multi-walled titanate nanotubes with diameters of about 8.26 nm (**Figure S3b**).

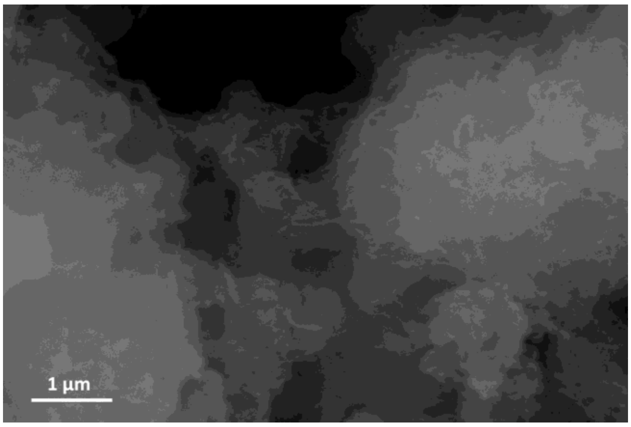

Figure S2. (a) SEM of TiNts

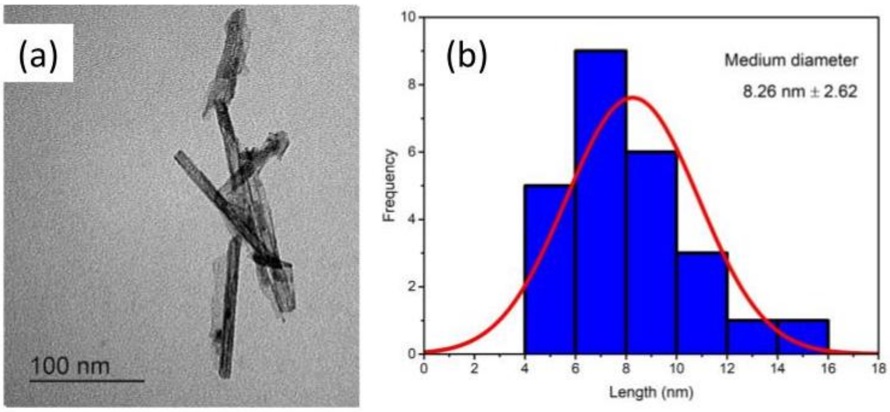

Figure S3. (a) TEM images and (b) distribution histogram of outer diameters of TiNts.

4. Excited-State Lifetime Analysis.

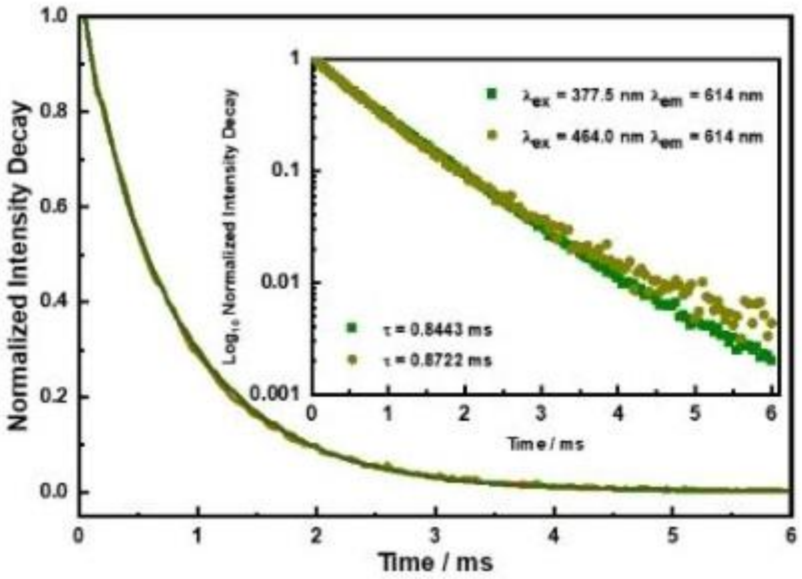

Figure S4. Luminescence decay curves of TiNts/Eu.

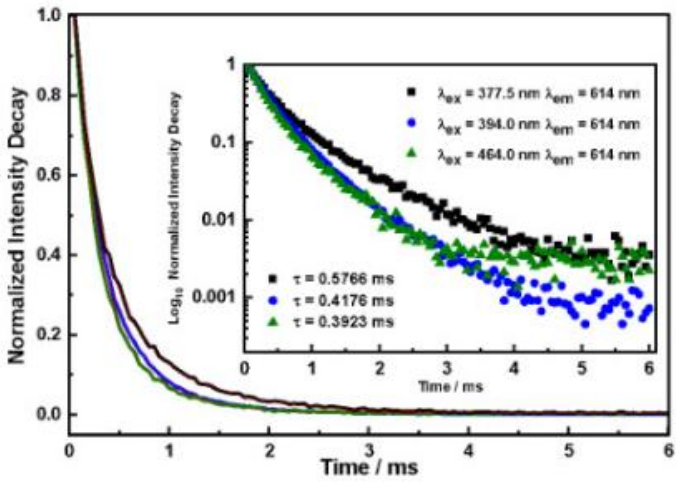

Figure S5. Luminescence decay curves of TiNts[Eu(tta)$_3$phen]

5. Photostability evaluation

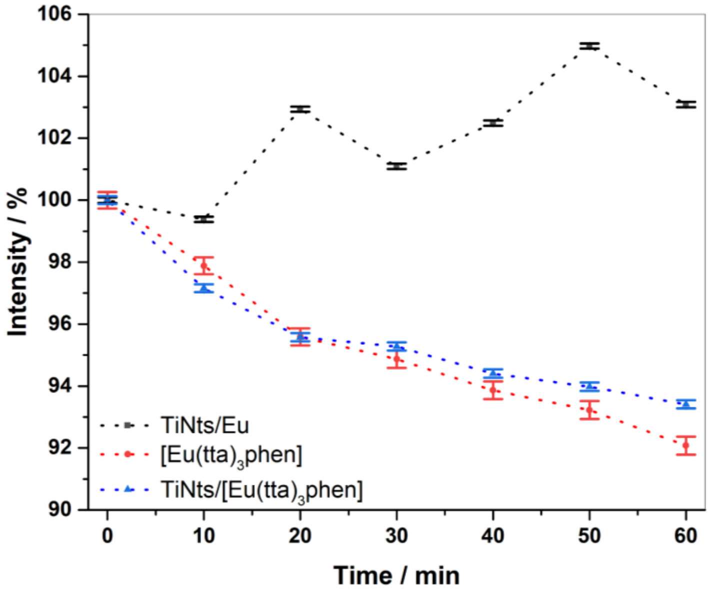

Figure S6. The photostability of the TiNts/Eu, TiNts[Eu(tta)3phen] and [Eu(tta)3phen]

6. Antibacterial activity assays

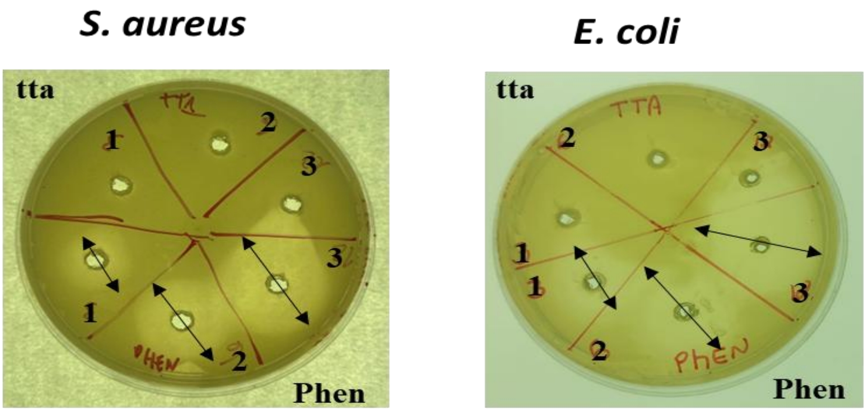

Figure S7. Antimicrobial activity of tta and phen to inhibit the growth of d *S. aureus and E. coli* ***Note:*** 1 (3 mg mL^{-1}), 2 (6 mg mL^{-1}), 3 (12 mg mL^{-1}). All tests were performed in duplicate for each bacterium.

REFERENCES

1. Wu, H.X.; Cao, W.M.; Wang, J.; Yang, H.; Yang, S.P. Coating multi-walled carbon nanotubes with rare-earth complexes by an in situ synthetic method. *Nanotechnology*, 2008, *19*(34), 345701.
